# Development of Interventions to Support Provincial Implementation of the Baby-Friendly Initiative: A Study Protocol

**DOI:** 10.3390/nursrep13040143

**Published:** 2023-12-13

**Authors:** Britney Benoit, Christine Cassidy, Marsha Campbell-Yeo, Doris Gillis, Sara Kirk, S. Meaghan Sim, Michelle LeDrew, Sally Loring, Gail Tomblin Murphy, Annette Elliott Rose, Claire Betker, Leanne MacKeen, Lindsay Arseneau, Kim Shebib, Trudy Reid, Ripu Daman

**Affiliations:** 1 Rankin School of Nursing, Faculty of Science, St. Francis Xavier University, Antigonish, NS B2G 2W5, Canada; lindsay.arseneau@gmail.com (L.A.); rdaman@stfx.ca (R.D.); 2 School of Nursing, Faculty of Health, Dalhousie University, Halifax, NS B3H 4R2, Canada; ccassidy@dal.ca (C.C.); marsha.campbell-yeo@dal.ca (M.C.-Y.); 3 Human Nutrition Department, Faculty of Science, St. Francis Xavier University, Antigonish, NS B2G 2W5, Canada; dgillis@stfx.ca; 4 School of Health & Human Performance, Faculty of Health, Dalhousie University, Halifax, NS B3H 4R2, Canada; sara.kirk@dal.ca; 5 Research, Innovation, & Discovery, Nova Scotia Health, Halifax, NS B3S 0H6, Canada; meaghan.sim@nshealth.ca (S.M.S.); gail.tomblinmurphy@nshealth.ca (G.T.M.); 6 Breastfeeding Committee for Canada, Glen Margaret, NS B3Z 3H8, Canada; bfiprojectdirector@bccbfi.com (M.L.); treasurer@breastfeedingcanada.ca (S.L.); 7 IWK Health, Halifax, NS B3K 6R8, Canada; annette.elliottrose@iwk.nshealth.ca; 8 National Collaborating Centre for Determinants of Health, St. Francis Xavier University, Antigonish, NS B2G 2W5, Canada; cbetker@stfx.ca; 9 Reproductive Care Program of Nova Scotia, Halifax, NS B3H 1Y6, Canada; leanne.mackeen@iwk.nshealth.ca; 10 Public Health, Nova Scotia Health, Halifax, NS B3S 1B8, Canada; kim.shebib@nshealth.ca (K.S.); reidtrudy@gmail.com (T.R.)

**Keywords:** Baby-Friendly Initiative, breastfeeding, implementation, barriers, facilitators, qualitative, Theoretical Domains Framework, Behaviour Change Wheel

## Abstract

Breastfeeding is internationally recognized as the optimal form of infant nutrition. The Baby-Friendly Initiative (BFI) is an evidence-informed program that leads to improved breastfeeding outcomes. Despite the benefits of breastfeeding, Nova Scotia has one of the lowest breastfeeding rates in Canada. Additionally, only two birthing hospitals in the province have BFI designation. We aim to address this gap using a sequential qualitative descriptive design across three phases. In Phase 1, we will identify barriers and facilitators to BFI implementation through individual, semi-structured interviews with 40 health care professionals and 20 parents. An analysis of relevant policy and practice documents will complement these data. In Phase 2, we will develop implementation interventions aimed at addressing the barriers and facilitators identified in Phase 1. An advisory committee of 10–12 administrative, clinical, and parent partners will review these interventions. In Phase 3, the interventions will be reviewed by a panel of 10 experts in BFI implementation through an online survey. Feedback on the revised implementation interventions will then be sought from 20 health system and parent partners through interviews. This work will use implementation science methods to support integrated and sustained implementation of the BFI across hospital/community and rural/urban settings in Nova Scotia. This study was not registered.

## 1. Introduction

Breastfeeding is a foundational practice that supports population health through protection of infants and mothers from infection and chronic disease, prevention of obesity, development of positive attachment, and promotion of individual, household, and community food security [1,2,3,4,5,6]. Universally recognized as the optimal form of infant nutrition, international guidelines recommend exclusive breastfeeding for the first six months with continuation for two years and beyond [2,3].

Despite the health-promoting benefits of breastfeeding, the province of Nova Scotia has one of the lowest breastfeeding rates in Canada [7,8], with only 26.8% of infants being exclusively breastfed to six months of age (compared to 34.5% nationally) [8]. Nova Scotia is a small province in Eastern Canada with a population of fewer than one million people [9]. Perinatal care is delivered by two health service organizations: IWK Health and Nova Scotia Health. IWK Health is the only tertiary referral perinatal center in Nova Scotia, with an annual birth rate of approximately 4500 infants [10]. Nova Scotia Health provides pregnancy, birth, and postpartum care in community hospitals across the entire province through the Women & Children’s Health program [11] for approximately 3500 births per year [12]. Although 89% of Nova Scotian women initiate breastfeeding in hospital, only 26.8% meet recommendations of exclusively breastfeeding for six months (compared to pan-Canadian rates of 91.1% and 34.5%, respectively) [8]. Intersections of the social determinants of health play an integral role in breastfeeding practice and resultant outcomes. Data from the Canadian Community Health Survey demonstrate that single mothers, mothers who have lower levels of either income or education, and racialized mothers are less likely to start and continue breastfeeding [7,8].

The Baby-Friendly Initiative (BFI) [2,3,13] is an internationally recognized breastfeeding promotion program, and adoption of the BFI leads to increased breastfeeding initiation, exclusivity, and duration at both hospital and population levels across diverse cultural contexts [3,14,15]. The BFI includes the Ten Steps for Successful Breastfeeding, which aim to optimize breastfeeding outcomes through enhanced breastfeeding knowledge, support, and mother-infant contact [2,13]. An example of one of the Ten Steps includes ensuring that all staff, health care providers, and volunteers have the knowledge and skills to support breastfeeding [16]. Organizations can obtain “Baby-Friendly” designation through successful implementation of the Ten Steps and compliance with the International Code of Marketing of Breast Milk Substitutes [2,13] (which provides policy guidelines and restrictions regarding marketing of breast milk substitutes such as infant formula).

Implementation of the BFI is a complex evidence-based change process. Previous research has identified that BFI implementation is challenged by a lack of government and health system support, commitment, and accountability; poor health service integration and communication; cultural infant feeding norms; inadequate health care provider education; socioeconomic disparity; and the negative impact of the infant formula industry [14,17]. Implementation research recommends the use of a context-focused, theory-informed approach to identify barriers at the local context, tailor interventions to address the barriers, and evaluate the effectiveness of implementation strategies [18]. This theory-informed approach is needed to facilitate BFI implementation, adoption, impact, and sustainability in Nova Scotia [19].

The timing is right to support BFI implementation in Nova Scotia. There is presently a commitment to province-wide BFI implementation to promote, protect, and support breastfeeding [20,21,22]. IWK Health received Baby-Friendly designation in 2018 and the Aberdeen Hospital (part of Nova Scotia Health) received Baby-Friendly status in 2020, making them the only institutions in the province with this status. This leaves remaining acute care hospitals in which perinatal services are delivered and public health offices that are working toward Baby-Friendly status but do not presently have this designation. Nova Scotia Public Health has committed to working with partners for BFI designation [23]. The Breastfeeding Committee for Canada (BCC) selected three hospitals in Nova Scotia (Aberdeen, South Shore Regional, Cape Breton Regional) as part of a national BFI quality improvement collaborative in which hospital teams have dedicated leadership support and meet regularly to share improvement strategies and monitor progress (initiated: 2020; ending: 2023; funded by the Public Health Agency of Canada) [20]. This quality improvement support includes one-on-one coaching from quality improvement and BFI experts; webinars and workshops to support teams; action periods where teams implement care changes; and leadership and parent partner networks [20]. Capitalizing on current commitment and initiatives, and comprising a natural experiment whereby some hospitals have adopted BFI where others have not, this research represents a strategic and timely partnership (BCC, Nova Scotia Health [NSH], IWK Health [IWK], Reproductive Care Program of Nova Scotia [RCP]) to characterize implementation processes in acute and community care contexts at different phases of BFI implementation, identify barriers and facilitators to BFI, and develop interventions to support province-wide BFI adoption.

## 2. Materials and Methods

### 2.1. Aim

Building on our previous work on BFI [17] and designing implementation strategies [24], the objective of this research is to support integrated and sustained implementation of the BFI in health organizations providing perinatal and infant care in Nova Scotia to optimize provincial breastfeeding outcomes. To achieve this objective, we will:

1. Identify barriers and facilitators to implementing the BFI in perinatal and infant care in Nova Scotia;
2. Develop theory-informed, contextually relevant implementation interventions for supporting BFI implementation and designation across perinatal and infant care contexts in Nova Scotia;
3. Determine the appropriateness and feasibility of the developed implementation interventions to support BFI implementation and designation across perinatal and infant care contexts in Nova Scotia.

### 2.2. Design

We will use a sequential qualitative descriptive design [25,26,27] guided by the Theoretical Domains Framework (TDF) [28,29] and the Behaviour Change Wheel (BCW) [30]. Barriers and facilitators that we identify will be used to develop tailored interventions to support BFI implementation. The TDF is an integrated framework that provides a guide for implementation studies [28,29,31]. Previous research has used the TDF to identify barriers to and facilitators of the use of evidence in various health care settings [29,30,32]. The BCW is a systematic intervention design guide that pairs with the TDF to develop tailored implementation interventions [30]. Our research team has successfully used these methods in implementation studies in pediatric care [33,34] and BFI [17]. We will employ multiple methods, including (a) qualitative interviews, (b) document analysis methods, and (c) intervention development methods in three phases to achieve our aims.

### 2.3. Setting

Our setting includes regional hospitals and community-based contexts providing perinatal and infant care across Nova Scotia, Canada. We will engage IWK Health and the Aberdeen Hospital (to identify facilitators that contributed to success in obtaining Baby-Friendly designation) and sites across NSH’s four health management zones (Central, Eastern, Northern, Western) to capture diverse care contexts at different stages of achieving Baby-Friendly status.

### 2.4. Phase 1: Identify Barriers and Facilitators to Implementing the BFI in Perinatal and Infant Care in Nova Scotia

We will apply a qualitative descriptive design [26,35], including one-on-one semi-structured interviews and document analyses, to develop a comprehensive understanding of provincial barriers to and facilitators of BFI implementation and designation.

#### 2.4.1. Qualitative Interviews

A stratified purposive sampling approach [29,36] will be used to recruit hospital- and community-based clinicians (e.g., acute care nurses, public health nurses, acute and primary care physicians and nurse practitioners, midwives, obstetricians, neonatologists), clinical leaders (e.g., lactation consultants, program clinical leads), administrators and policy-makers (e.g., program managers, directors, public health medical officers of health), and parents who have given birth in Nova Scotia and accessed perinatal or infant services in the last 24 months (parent participants). We will recruit approximately 60 participants: 20 parent participants (5 each per health management zone) and 40 health system participants (10 each per heath management zone) [36]. Qualitative sampling criteria [37] will be used in recruitment to ensure that participants represent diverse perspectives on BFI implementation in Nova Scotia. These criteria promote consideration of clarity, similarities, and differences across the data to guide focused recruitment of additional participants. All participants will receive a $20.00 gift card for participation. We will leverage our research team partners and networks to identify health system participants. An invitation to participate will be sent in a recruitment email outlining study details. To recruit parent participants, we will circulate recruitment posters over social media and place posters in perinatal care areas across Nova Scotia. Our research team includes leaders from the National Collaborating Centre for Determinants of Health (NCCDH). They will lend expertise to support sampling of participants with diverse and intersecting identities across sex, gender, race, ethnicity, socioeconomic status, im/migration status, sexual orientation, ability, and geography.

Virtual, one-on-one, semi-structured interviews with each consenting participant will be completed (through Zoom or telephone, based on participant preference and accessibility). Interview data will be analyzed using NVivo (Version 12) [38]. The TDF domains [28,29] (Supplementary S1) will be used to develop the semi-structured interview guide and facilitate analysis of participant interviews. Audio-recorded interviews will be transcribed to facilitate analysis. Transcripts will be coded using inductive–deductive qualitative content analysis [25,39]. First, two independent researchers will deductively code [25,39] data into the domains of the TDF [28]. Second, themes and sub-themes of barriers and facilitators will be generated through inductive analysis [25,39]. We will use a variety of strategies to promote trustworthiness for qualitative [40,41] implementation [42] studies, including clearly reporting the analysis procedure [42,43], reporting participant characteristics and the study context [36,40,42,43], and having participants verify the analysis [40,41,43]. Data from this diverse group of participants will support identification of interventions to support implementation of the BFI in acute and community care contexts across the province.

#### 2.4.2. Key Document Analysis

Document analysis is a systematic procedure for reviewing or evaluating documents as an additional source of information in qualitative studies to elicit meaning, gain understanding, identify context, and develop empirical knowledge [44]. Documents can provide data on the health system context in which BFI implementation is taking place; suggest questions or areas of focus for qualitative interviews; provide supplementary data on barriers to and facilitators of BFI implementation; and corroborate findings from the qualitative interviews. Document analyses are often used in combination with other qualitative research methods to seek convergence in understanding a phenomenon (like implementation of complex interventions within health systems) through the use of multiple sources of information [44]. Purposive sampling of relevant provincial and institutional policy documents (e.g., provincial infant feeding policies [21]), policy implementation materials, reports (e.g., institutional BFI or breastfeeding committee reports, evaluation reports [45]), and any other recommended contextual documents (identified through participant interviews) relevant to BFI implementation in the province will be completed. Inclusion criteria will be policy and practice documents published in English relevant to breastfeeding promotion and BFI implementation in Nova Scotia. Documents regarding breastfeeding care and BFI relevant to diverse disciplines (e.g., dietetics, medicine, midwifery, nursing, psychology, social work) and contexts (e.g., acute care, primary care, public health) will be considered eligible. Each document will be reviewed by two research team members using an iterative process of skimming, reading, and interpreting to code and categorize document data [44]. We will use the TDF domains (Supplementary S1) [29] and Behaviour Change Wheel intervention functions (Supplementary S2) [30] to categorize coded data to facilitate triangulation of findings [44,46] with study interviews. The document analysis will synthesize the breadth of data on historical and current policies and practices relevant to BFI implementation in Nova Scotia. As it will be completed concurrently with interviews, it will also be used to guide key areas for exploration with participants.

#### 2.4.3. Interview and Document Analysis Integration

Data collected from the qualitative interviews will be triangulated with document analysis data to examine convergence, divergence, and inconsistencies between qualitative interview and document analysis findings using a triangulation protocol [44,46,47]. The TDF [28,29] is being used to code and characterize data across interview and document analysis components. Therefore, we will develop a tabular triangulation coding matrix based on the TDF domains (Supplementary S1) to document and display findings from both the participant interviews and document analysis. Through a review of this triangulation matrix, we will identify convergence, divergence, and inconsistencies across both study components to clearly understand the interconnectedness of the data.

#### 2.4.4. Phase 1 Success Indicators

A synthesis of relevant key documents will be completed, and participant interviews will be completed with 60 health system and parent partners. The research team will have completed data analysis and integration and will have a comprehensive understanding of the current policy and practice contexts and associated barriers to and facilitators of BFI implementation in Nova Scotia.

### 2.5. Phase 2: Develop Theory-Informed, Contextually Relevant Implementation Interventions for Supporting BFI Implementation and Designation across Perinatal and Infant Care Contexts in Nova Scotia

Building on our findings from Phase 1, we will develop implementation interventions to support identified facilitators and overcome identified barriers to BFI implementation. To do this, our team will utilize the Behaviour Change Wheel (BCW) [30]. We will take a two-step approach to intervention design.

#### 2.5.1. Phase 2(a) Intervention Mapping

We will begin by reviewing Phase 1 findings alongside the BCW [30]. The BCW includes nine evidence-based intervention functions (e.g., education, training, enablement; Supplementary S2) that can be effective for addressing the behaviours identified in Phase 1. The barriers and facilitators identified in study Phase 1 will be mapped onto these intervention functions. We will then map the intervention functions onto specific BCW behaviour change techniques, which are the active components of the intervention functions that change behaviour [30]. From this phase, we will have tailored interventions that are specifically targeted toward the barriers and facilitators across contexts from study Phase 1.

#### 2.5.2. Phase 2(b) Advisory Committee Meetings

An advisory committee of 10–12 administrative, clinical, and parent partners will be formed. Advisory committee members will consist of participants from Phase 1 interviews who agreed to be contacted regarding potential participation in the Phase 2 advisory committee. Committee members will be purposively invited to promote inclusion of intersecting representations of identity across sex, gender, race, ethnicity, socioeconomic status, im/migration status, sexual orientation, ability, and geography. The advisory committee will include a minimum of two parents. We will hold two, 3 h meetings with the advisory committee. Parent partners will be provided $150/session [48]. The meeting will begin with an overview of findings from the previous study phases (Phase 1 and 2a). This will be followed by a critical review and discussion of the findings guided by the APEASE criteria (Affordability, Practicability, Effectiveness and cost-effectiveness, Acceptability, Side-effects and safety, Equity) [30]. Details of the discussion will be documented by a member of the research team [42]. Input from the advisory committee will help identify the relevance and feasibility of potential interventions, refine intervention details (e.g., content, intensity, duration), and strategize ways in which the interventions could be implemented across various health care contexts.

#### 2.5.3. Phase 2 Success Indicators

A package will be circulated to the study team that details the implementation interventions that were mapped onto the identified barriers and facilitators from Phase 1. Two advisory committee meetings will be held during which details of the implementation interventions will be discussed and refined. Following completion of Phase 2, we will have co-designed, theoretically informed implementation interventions to support BFI implementation to undergo pre-pilot testing in study Phase 3.

### 2.6. Phase 3: Determine the Appropriateness and Feasibility of the Implementation Interventions to Support BFI Implementation and Designation in Nova Scotia

To ensure that the developed implementation interventions from Phase 2 are appropriate and meet the needs of the diverse provincial health system and community contexts aiming to implement the BFI, we will complete preliminary pre-pilot testing of the developed implementation interventions from Phase 2. This pre-pilot testing will be completed in two iterations: Phase 3(a) expert review of the implementation interventions and Phase 3(b) partner feedback on the implementation interventions and proposed implementation into care contexts.

#### 2.6.1. Phase 3(a) Expert Review

The developed implementation interventions will be reviewed by a panel of ten experts in BFI implementation. These experts will be purposively selected to include health professionals and/or implementation scientists with specific expertise in BFI implementation in the Canadian context. These experts will be recruited through research team partnerships with the BCC and the RCP. Selected experts will be sent a recruitment email with study details and an invitation to participate. Participating individuals will receive documentation describing the refined implementation interventions from Phase 2(b) and will be asked to complete an online survey [49] collecting information related to the appropriateness and feasibility of the implementation interventions. The APEASE criteria [30,32] will be used to structure open-ended survey questions. Survey responses will be summarized through inductive–deductive qualitative content analysis using the APEASE criteria to deductively frame participant responses [25,30]. Implementation interventions will be modified based on expert recommendations and all decisions will be documented based on intervention development guidelines [42]. Following discussion and consensus from all research team members, a second iteration of the implementation interventions will be developed to be evaluated by key partners in Phase 3(b).

#### 2.6.2. Phase 3(b) Partner Feedback

Feedback on the second iteration of the implementation interventions will be sought from 20 health system and parent partners through semi-structured qualitative interviews to ensure applicability and feasibility for different contexts. Using stratified purposive sampling methods [36] (as happened for the initial qualitative interviews), we will recruit a new and unique group of hospital- and community-based clinicians, clinical leaders, administrators and policy-makers, and parents. We will strategically recruit participants to ensure diverse and intersecting representations of identity across sex, gender, race, ethnicity, socioeconomic status, im/migration status, sexual orientation, ability, and geography. All participants will be offered a $20.00 gift card as an honorarium for participation. The APEASE criteria [30] will be used to develop the semi-structured interview guide and support analysis of participant interviews. Transcriptions of audio-recorded interviews will be analyzed using an inductive–deductive qualitative content analysis [25,39]. First, two reviewers will deductively categorize [25,39] participant responses in the interview data based on each of the APEASE criteria categories. Second, inductive qualitative content analysis will be utilized to generate categories of salient implementation considerations within each of the APEASE criteria categories [25,39].

#### 2.6.3. Phase 3 Success Indicators

We will have completed and analyzed survey and interview data and modified the BFI implementation interventions based on expert and key partner feedback. Following completion of this phase, we will have evidence-informed implementation interventions that have undergone pre-pilot testing, demonstrated face validity, and are specifically tailored to diverse clinical and community contexts.

## 3. Discussion

### 3.1. Challenges and Mitigation Approaches

Based on our experience conducting implementation research in breastfeeding and BFI promotion in clinical and community organizations, there are three potential challenges we have identified. We have embedded mitigation approaches into our research procedures to ensure success.

#### 3.1.1. Scheduling and Availability

Finding time for interviews and advisory committee meetings with diverse partners can be challenging due to competing priorities and scheduling differences. We will employ flexible scheduling based on participant preferences; scheduling on continuing education days or program quality improvement meetings/staff meetings; and scheduling in the morning or evening around shifts. We will have research staff dedicated to scheduling and facilitating participant interviews who will employ these strategies.

#### 3.1.2. Participant Recruitment and Representation

We have participant recruitment support through our research team, which includes provincial health systems’ partners (IWK, NSH), strategic perinatal care programs and breastfeeding promotion committees (RCP, BCC), a parent partner (LA), and support from the Maritime Strategy for Patient Oriented Research (SPOR) Support Unit (MSSU). Furthermore, we have commitment from the NCCDH to support recruitment and engagement of participants from equity-deserving groups in interviews and advisory committee meetings. We additionally will be providing all participants who take part in study interviews a gift card as an honorarium and paying parent contributors on our advisory committee consistent with patient partner compensation guidelines [48].

#### 3.1.3. COVID-19 and Impact on Research

Our study team has experience conducting virtual interviews and meetings using Zoom and we have the capacity to complete the entire study remotely. Furthermore, participants for study interviews may be experiencing additional/shifting workload demands and commitments as part of the health systems’ COVID-19 response. As such, we have dedicated long time blocks to conduct study interviews and account for competing priorities.

### 3.2. Patient Engagement

Key patient and caregiver engagement guidelines [50,51] were used to develop our patient engagement plan. Patients will be engaged throughout this research in various ways to ensure their perspectives are well represented. A parent partner (LA) is an integral member of our research team. Parents are participants in study interviews and the study advisory committee, where they will provide key input on development, adaptation, and implementation of interventions to support BFI. Parents will be paid for their contributions [48]. Parents with diverse perspectives will be purposively engaged throughout this research to support understanding of the ways in which equity, diversity, inclusion, and accessibility influence breastfeeding and the BFI in Nova Scotia.

### 3.3. Sex, Gender, and Equity

Lack of awareness and acknowledgement of sex and gender inequities in health services are barriers to access and use and impede the processes by which policy-makers and care providers reduce gendered inequities in health [52]. Consideration of the ways in which sex, gender, and intersections in the social determinants of health impact health systems and services enables the development of more effective policies and practices that can better support BFI implementation [53] and facilitate costs savings to health systems [54]. A sex- and gender-based^+^ analysis [52] will be used to understand the intersecting influences of the social determinants of health on BFI implementation. We will consciously recruit and interview persons experiencing health and social inequities across all phases of this work and will explore patterns across subgroups based on sex, gender, race, ethnicity, socioeconomic status, im/migration status, sexual orientation, ability, and geography. Knowledge dissemination will include findings related to the role of the social determinants of health to support the consideration in health service planning and decision-making related to BFI implementation. Our team includes experts in the social determinants of health, gender, and health equity (including partnership with the NCCDH).

### 3.4. Knowledge Translation

Intentional engagement of knowledge users, including key heath systems’ partners, clinical care programs, and parents as team members and collaborators, ensures generation of relevant, high-quality evidence. It also provides an excellent infrastructure for rapid dissemination of findings into policy, practice, and clinical care. Knowledge translation initiatives will be ongoing throughout this research, including integrated and end-of-grant knowledge translation (KT).

#### 3.4.1. Integrated KT

We will regularly engage with all research team members, who include key knowledge users, through virtual team meetings to ensure opportunity for input and feedback through all stages of the research. The findings of each study phase may be immediately relevant to partners. Therefore, interim research summary reports will be circulated through email, existing webinars, and continuing education infrastructure established through our team members and collaborators (e.g., RCP, BCC, NCCDH, MSSU, IWK, NSH).

#### 3.4.2. End-of-Grant KT

To support health system planning regarding BFI implementation, we will generate an end-of-grant summary report of key findings to be integrated into policy and clinical practice to be disseminated through our health systems’ partners and end-users. Our findings will be disseminated to breastfeeding promotion and implementation sciences researchers in Canada and internationally through conferences and will be published in open access journals to enhance accessibility and potential for global reach and impact.

## Data Availability

Data will not be made available due to research ethics restrictions.

## Supplementary Materials

The following supporting information can be downloaded at: https://www.mdpi.com/article/10.3390/nursrep13040143/s1, Supplementary S1: Theoretical Domains Framework domains with definitions and component constructs. Adapted from: Atkins et al. (2017). A guide to using the TDF of behaviour change to investigate implementation problems. *Implementation Science*, 12(77), 1–18 [29]. Supplementary S2: Behaviour Change Wheel with definitions and intervention and policy examples. Adapted from: Michie et al. (2011). The behaviour change wheel: a new method for characterising and designing behaviour change interventions. *Implementation Science*, 6(42), 1–11. [30].

## Public Involvement Statement

We have a dedicated parent partner as a member of our research team (LA) who will represent parent and family perspectives. Parents will provide their perspectives through study interviews and will be asked to provide feedback on the analysis of interview data. Parents will also participate on our advisory committee, where they will contribute to discussion regarding the adaptation and implementation of interventions to support BFI.

## Guidelines and Standards Statement

The findings of this study will be drafted against the “Guidance for reporting intervention development studies in health research (GUIDED): An evidence-based consensus study” checklist [42].
