# Supplementary material for: Development of Interventions to Support Provincial Implementation of the Baby-Friendly Initiative: A Study Protocol
Source: Nurs Rep. 2023 Dec 13;13(4):1731–41. doi: 10.3390/nursrep13040143 (PMC10745531; doi:10.3390/nursrep13040143)
Supplement: Supplementary file 1 [file nursrep-13-00143-s001.zip › nursrep-2588851-supplementary.pdf]

Supplementary S1

Theoretical Domains Framework domains with definitions and component constructs

Adapted from: Atkins et al., (2017). A guide to using the TDF of behaviour change to investigate implementation problems. *Implementation Science*, 12(77), 1-18.

Component	Definition	Component Constructs
**Knowledge**	An awareness of the existence of something	Knowledge<br>Knowledge of condition Scientific rationale<br>Procedural knowledge<br>Knowledge of environment
**Skills**	An ability or proficiency acquired through practice	Skills<br>Skill development<br>Competence<br>Ability<br>Interpersonal skills<br>Practice<br>Skill assessment
**Social/professional role identity**	A coherent set of behaviours and displayed personal qualities of an individual in a social of work setting	Professional identity<br>Professional role<br>Social identity<br>Identity<br>Professional boundaries<br>Professional confidence<br>Group identity<br>Leadership<br>Organizational commitment
**Beliefs about capabilities**	Acceptance of the truth, reality of validity about an ability, talent or facility that a person can put to constructive use	Self-confidence<br>Perceived competence<br>Self-efficacy<br>Perceived behavioural control

		Beliefs<br>Self-esteem<br>Empowerment<br>Professional confidence
**Optimism**	The confidence that things will happen for the best of that desired goals will be attained	Optimism<br>Pessimism<br>Unrealistic optimism<br>Identity
**Beliefs about consequences**	Acceptance of the truth, reality, or validity about outcomes of a behaviour in a given situation	Beliefs<br>Outcome expectancies<br>Characteristics of outcomes<br>Expectancies<br>Anticipated regret<br>Consequences
**Reinforcement**	Increasing the probability of a response by arranging a dependent relationship, or contingency, between the response and a given stimulus	Rewards<br>Incentives<br>Punishment<br>Consequences<br>Reinforcement<br>Contingencies<br>Sanctions
**Intentions**	A conscious decision to perform a behaviour or a resolve to act in a certain way	Stability of intentions<br>Stages of change model<br>Transtheoretical model and stages of change
**Goals**	Mental representation of outcomes or end states that an individual wants to achieve	Goals (distal/proximal)<br>Goal priority<br>Goal/target setting<br>Goals (autonomous/controlled)<br>Action planning<br>Implementation intention

**Memory, attention, and decision processes**	The ability to retain information, focus selectively on aspects of the environment and choose between two or more alternatives	Memory<br>Attention<br>Attention control<br>Decision making<br>Cognitive overload/tiredness
**Environmental context and resources**	Any circumstance of a person's environment that discourages or encourages the development of skills and abilities, independence, social competence, and adaptive behaviour	Environmental stressors<br>Resources/material resources<br>Organizational culture/climate<br>Salient events/critical incidents<br>Person x environment interaction<br>Barriers and facilitators
**Social influences**	Those interpersonal processes that can cause individuals to change their thoughts, feelings, or behaviours	Social pressure<br>Social norms<br>Group conformity<br>Social comparisons<br>Group norms<br>Social support<br>Power<br>Intergroup conflict<br>Alienation<br>Group identity<br>Modelling
**Emotion**	A complex reaction pattern involving experiential, behavioural, and physiological elements by which the individual attempts to deal with a personally significant matter or event	Fear<br>Anxiety<br>Affect (positive/negative)<br>Stress<br>Depression<br>Burn out
**Behavioural regulation**	Anything aimed at managing or changing objectively observed or measured actions	Self-monitoring<br>Breaking habit<br>Action planning

Supplementary S2

Behaviour Change Wheel with definitions and intervention and policy examples

Adapted from: Michie et al., (2011). The behaviour change wheel: A new method for characterising and designing behaviour change interventions. *Implementation Science*, 6(42), 1-11.

Intervention	Definition	Examples
**Education**	Increasing knowledge or understanding	Providing information on benefits of breastfeeding for pain management
**Persuasion**	Using communication to stimulate action	Using posters and clinical practice prompts to encourage placing infants in skin-to-skin contact during procedures
**Incentivisation**	Creating expectation of award	Providing awards/recognition to clinicians who support parent-infant skin-to-skin contact during painful procedures
**Coercion**	Creating expectation of punishment or cost	Using Adverse Events Management System reporting when infant painful procedures completed with no intervention
**Training**	Imparting skills	Training on completing blood work when babies are in skin-to-skin contact
**Restriction**	Using rules to increase the target behaviour by reducing the opportunities to engage in competing behaviour	Restricting use of pharmacologic strategies to promote parent-led interventions
**Environmental restructuring**	Changing the physical or social context	Putting in place necessary physical infrastructure (stools) to enable clinicians to complete procedures with ergonomic safety when infants are being held by parent
**Modelling**	Providing an example for people to aspire to or imitate	Using respected clinical leaders to model use of breastfeeding during painful

		procedures
**Enablement**	Increasing means/reducing barriers to increase capability or opportunity[a]	Having sufficient staff to support use of parental pain-relieving interventions; ensuring families present during procedures

[a]Capability beyond having required education and training; opportunity beyond environmental restructuring
